# A triple-classification for differentiating renal oncocytoma from renal cell carcinoma subtypes and CK7 expression evaluation: a radiomics analysis

**DOI:** 10.1186/s12894-022-01099-0

**Published:** 2022-09-12

**Authors:** Ziyang Yu, Jie Ding, Huize Pang, Hongkun Fang, Furong He, Chenxi Xu, Xuedan Li, Ke Ren

**Affiliations:** 1 School of Medicine, Xiamen University, Xiamen, Fujian Province China; 2 Department of Radiology, First Affiliated Hospital of China Medical University, Shenyang, Liaoning Province China; 3 Radiology, Xiang’an Hospital of Xiamen University, Xiamen, China

**Keywords:** Renal cell carcinoma, Renal oncocytoma, Radiomics, Support vector machine, Cytokeratin 7

## Abstract

**Background:**

To investigate the value of computed tomography (CT)-based radiomics model analysis in differentiating renal oncocytoma (RO) from renal cell carcinoma subtypes (chromophobe renal cell carcinoma, clear cell carcinoma) and predicting the expression of Cytokeratin 7 (CK7).

**Methods:**

In this retrospective study, radiomics was applied for patients with RO, chRCC and ccRCC who underwent surgery between January 2013 and December 2019 comprised the training cohort, and the testing cohort was collected between January and October 2020. The corticomedullary (CMP) and nephrographic phases (NP) were manually segmented, and radiomics texture parameters were extracted. Support vector machine was generated from CMP and NP after feature selection. Shapley additive explanations were applied to interpret the radiomics features. A radiomics signature was built using the selected features from the two phases, and the radiomics nomogram was constructed by incorporating the radiomics features and clinical factors. Receiver operating characteristic curve was calculated to evaluate the above models in the two sets. Furthermore, Rad-score was used for correlation analysis with CK7.

**Results:**

A total of 123 patients with RO, chRCC and ccRCC were analyzed in the training cohort and 57 patients in the testing cohort. Subsequently, 396 radiomics features were selected from each phase. The radiomics features combining two phases yielded the highest area under the curve values of 0.941 and 0.935 in the training and testing sets, respectively. The Pearson’s correlation coefficient was statistically significant between Rad-score and CK7.

**Conclusion:**

We proposed a non-invasive and individualized CT-based radiomics nomogram to differentiation among RO, chRCC and ccRCC preoperatively and predict the immunohistochemical protein expression for accurate clinical diagnosis and treatment decision.

**Supplementary Information:**

The online version contains supplementary material available at 10.1186/s12894-022-01099-0.

## Introduction

Adult renal tumors were classified according to pathology, clinical epidemiology, and genetics by the World Health Organization (WHO) in 2016. One subset of adult renal tumors exhibits granular cytoplasm, among which the common types were renal oncocytoma (RO) and chromophobe renal cell carcinoma (chRCC) [1]. Both chromophobe renal cell carcinoma (chRCC) and RO originate from renal intercalated cells and account for 6–8% and 3–7% of all renal tumors, respectively [2]. In addition, clear cell carcinoma (ccRCC) is the most common renal neoplasm, the overlapping imageology features also make differentiation between RO and ccRCC challenging to a degree [3]. Despite various overlapping features, the varied physiological characteristics lead to disparate management and follow-up strategies [4]. Patients with RO usually require active surveillance because of the benign characteristic and excellent prognosis [5]. Conversely, chRCC is managed by partial nephrectomy, while radical resection is recommended for ccRCC [6]. Therefore, differential diagnosis of ccRCC, chRCC and RO is critical to making treatment strategy decisions.

Computerized tomography (CT), especially dynamic contrast-enhanced (DCE)-CT is the preferred and the most common non-invasive preoperative method for the diagnosis of renal lesions. However, radiologists still face challenges in differentiating chRCC from RO because of overlapping imaging manifestations [7]. Some biomarkers, such as delayed enhancement of central stellate scar, have been proposed for RO diagnosis. However, only 25–30% of RO patients may present a central scar in practice, resulting in a high false-negative rate [8]. Some studies have illustrated that approximately 20% of chRCC could also be manifested with similar CT imaging finding [9]. In addition, necrotic area within ccRCC could also appear as a central scar. Therefore, the diagnosis of benign RO may not be precise when a renal mass with a central scar is observed on CT images.

An accurate differentiation of renal tumours relies on the histochemistry of the sections and the characteristic morphological features. The advancements in other techniques, such as immunohistochemistry and electron microscopy, have facilitated the identification of subtle pathological characteristics. However, these are neither cost-effective nor easily available. Modern molecular biomarkers of tumors have been identified for customized diagnosis and targeted therapy. Cytokeratin 7 (CK7) is a low-molecular-weight cytokeratin, expressed in the urothelium and epithelia. Several studies have shown that CK7 is more readily expressed in chRCC than ccRCC and RO [10]. Moreover, it is involved in cell cycle progression and differentiation [11], which may contribute to accurate diagnosis and also be a potential therapeutic target in renal tumor subtypes [12].

Radiomics is a promising method that gathers mineable medical data from texture analysis [13]. It quantitatively analyzes the inherent heterogeneity of tumor lesions [14–16] and has been used as a clinical biomarker for prognosis or prediction in a broad research field [17, 18]. Several studies have confirmed that radiomics is not only valuable in evaluating renal tumours [19] but also in other oncological fields of urology [20]. In addition, recent studies also have demonstrated that multimodal imaging could help predict tumor staging and prognosis [21, 22]. However, previous studies lacked the interpretability of radiomics models, which led to skepticism about the underlying mechanisms of the radiomics features. In the current study, we explained our classifiers by Shapley additive explanations (SHAP) framework to increase their usability [23]. Currently, SHAP is the most recommended tool for model explanation. It assigns a weight value to each feature in the model. Then, the values for each prediction are calculated independently, and high absolute SHAP values indicate importance, whereas values close to zero indicate low usability. Thus, we hypothesized that the combination of radiomics features extracted from the two phases enhances the accurate diagnosis of the two renal tumor subtypes and the expression of CK7.

Therefore, the present study aimed to develop a non-invasive and interpretable nomogram combining CT radiomics features from corticomedullary phase (CMP) and nephrographic phase (NP) with clinical variables to differentiate between RO and renal cell carcinoma subtypes. In addition, we further investigate the correlation between the radiomics signature and CK7 index which may provide a promising molecular target for chRCC precise therapy.

## Materials and methods

### Patients

**Fig. 1:**
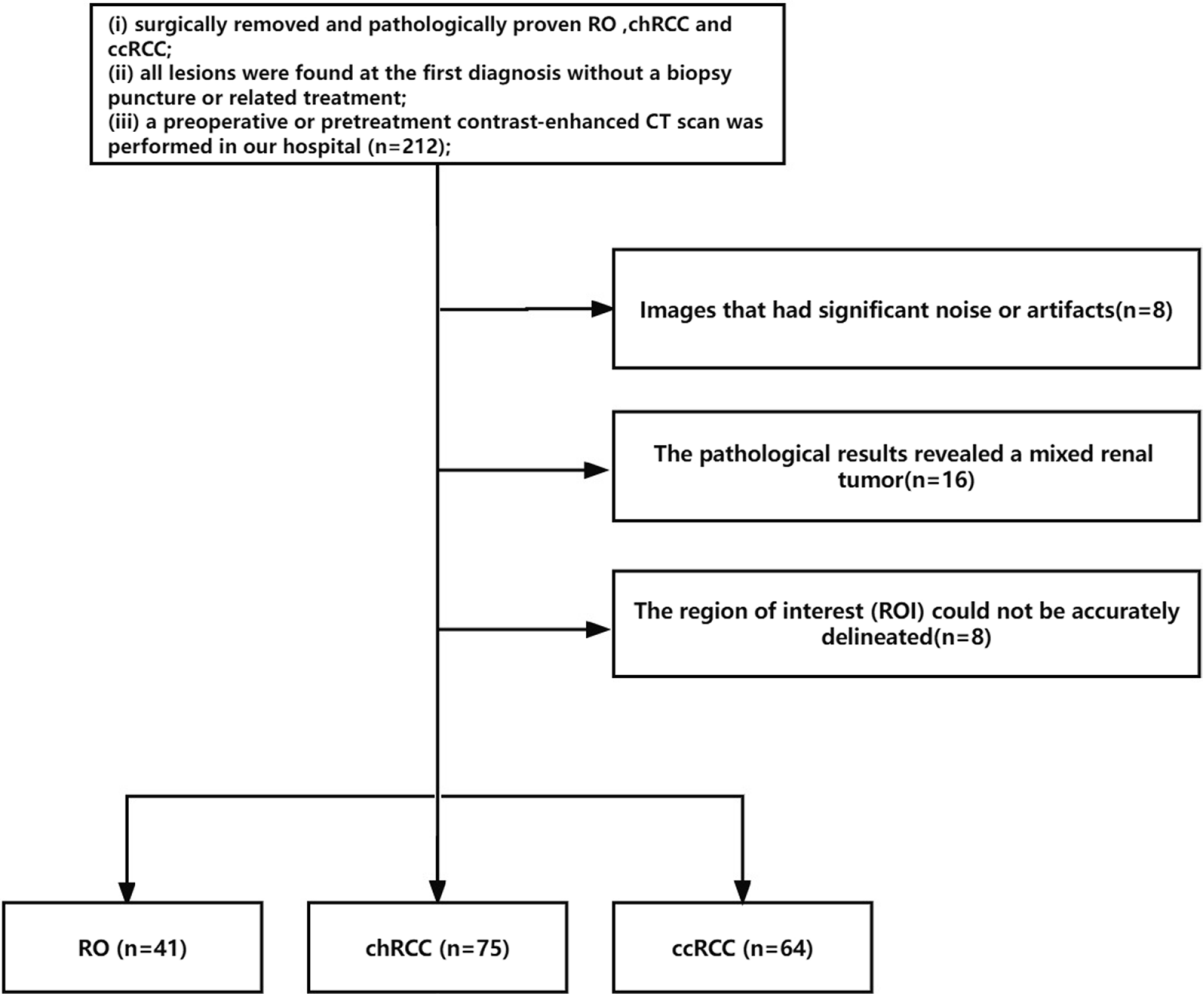
Flowchart illustrates patient recruitment

This retrospective study was approved by the institutional review board of the China Medical University, and the requirement for patient informed consent was waived. The enrolled patients had histologically proven ccRCC, chRCC or RO from January 1, 2013 to October 31, 2020 were collected from Picture Archiving and Communication System (PACS). The inclusion criteria were as follows: (i) surgically removed and pathologically proven ccRCC, chRCC and RO; (ii) all lesions were found at the first diagnosis without a biopsy puncture or related treatment; (iii) a preoperative or pretreatment contrast-enhanced CT scan was performed in our hospital; (iv) a renal function examination was performed in our hospital within one week after the contrast-enhanced renal CT scan. The exclusion criteria were as follows: (i) images that had significant noise or artifacts; (ii) pathological results revealed a mixed renal tumor; (iii) the lesion was < 1.0 cm, and the region of interest (ROI) could not be delineated accurately. The patient inclusion/exclusion criteria are presented in Fig. 1. The training cohort comprised patients from January 2013 to December 2019, and the independent testing cohort consisted of the patients between January and October 2020.

### CT image acquisition

All patients were scanned using a Philip Lightspeed 256-row CT machine with a tube voltage of 120 kV and a tube current of 100 mA. A nonionic contrast agent (containing 300 mg/mL iodine) was infused into the peripheral vein at 1.5 mL/kg infusion dose. Owing to the effect of weight on metabolism, the injection was completed within 25 s. The scan ranged from the diaphragm to the anterosuperior iliac spine with a thickness of 5 mm/layer. The CMP and NP scans were performed 25–30 s and 60–70 s after the contrast agent injection, respectively.

### Evaluation of CT features

Two abdominal radiologists with 5 and 10 years of experience, respectively, assessed the CT features blindly and independently: CT value difference were noted between CMP and NP enhancement, and finally, these values were averaged. The results were assessed by a senior physician (Xuedan Li, with > 30 years of experience in abdominal diagnosis).

### Tumor segmentation

**Fig. 2:**
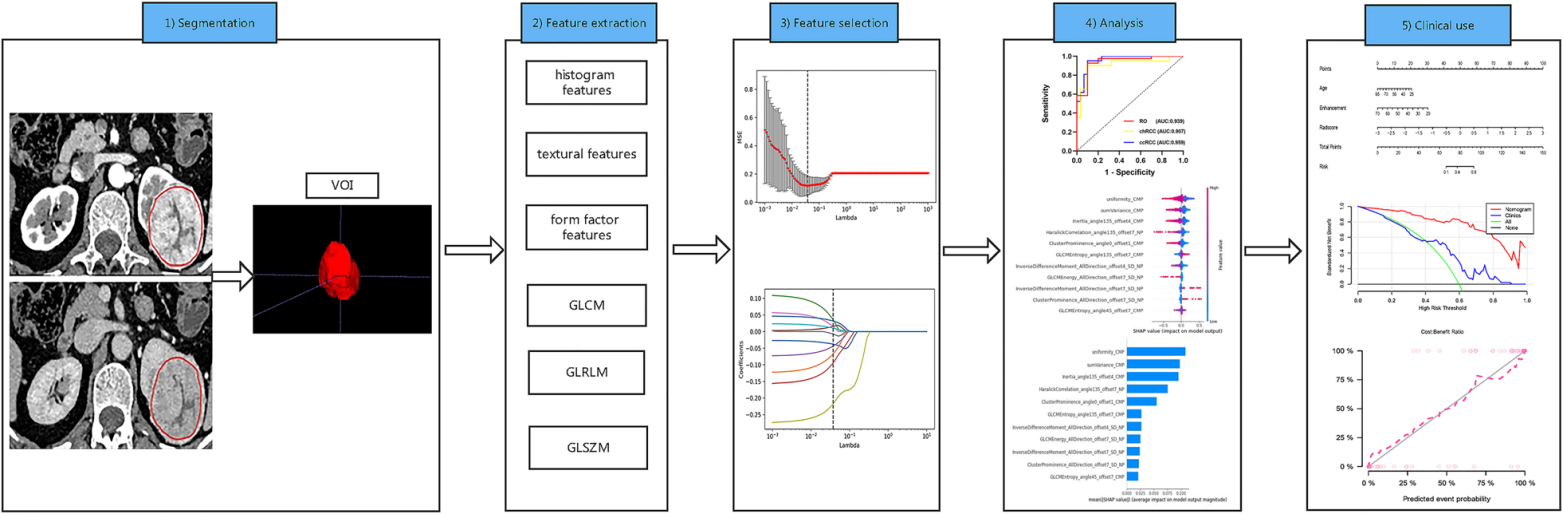
Workflow of radiomics methodology. (1) The example of tumor segmentation on the CT image of a cross-axial section. The contours were drawn slightly within the borders of the tumor. The tumor was segmented on both corticomedullary and nephrographic phase images, respectively. Thus, VOI was generated by a continuous layer of delineation. (2) Six types of radiomics features were analyzed via AK software. (3) LASSO was applied in the training set for feature selection. (4) The models were evaluated by ROC curve analysis. SHAP values were applied with the SVM models to transparentize the “black box.” (5) A nomogram that incorporates radiomics signature and clinical factors was constructed to provide a visual measure for customized evaluation, followed by decision curve analysis and calibration curve

The two radiologists drew the ROIs independently, and all the lesions were identified correctly by the senior physician. The radiologists were unaware of the diagnosis and blinded to the pathology results. To reduce the partial volume effect, the ROI was drawn carefully to encompass the visible lesion contour within the margins of the tumor on CMP and NP axial images using the software package ITK-SNAP version 4.11.0 (www.itk-snap.org), and the final volumes of interest (VOIs) were generated accordingly. An example of the manual segmentation process is shown in Fig. 2.

### Radiomics feature extraction and selection

All VOIs were imported into A.K. software version V3.0.0. R (Analysis Kit, GE Healthcare, China). The reproducibility of the extracted features was measured by intra-class correlation coefficients (ICCs). A total of 20 patients were selected randomly, and the inter-observer reproducibility was assessed by the two radiologists. Subsequently, the radiologist (Jie Ding) remarked the ROIs on these 20 patients after five days. Only the features with ICC > 0.80 were retained for the subsequent analysis. The extracted radiomics features were standardized into a normal distribution (z-scores) to avoid dimension bias.

To avoid redundant data, all radiomics features with good agreement of ICCs (> 0.8) from CMP and NP were analyzed by least absolute shrinkage and selection operator (LASSO). respectively, a method for feature selection for super-dimensional data. The tuned parameter λ was selected according to the smallest ten-fold cross-validation error score in the training set. The optimal parameters are listed in Additional file 1: Table S1.

### Classification and evaluation

Support vector machine (SVM) classifier with a radial basis function (RBF) kernel was used in our study for classification. The extracted radiomics features were standardized into a normal distribution (z-scores) to avoid dimension bias, and the parameter class-weight was set at “balanced” to avoid sample bias. Furthermore, to avoid model overfitting, the classifiers were constructed using ten-fold cross-validation in the training cohort based on the CMP, NP, and the CMP-NP combination. The parameters of classifiers were set according to their stability and best performance by “Grid Search CV” algorithm [24]. The SVM parameters are listed in Additional file 1: Table S1.

The performance of the classifiers was evaluated on the testing set independent of the training set. To evaluate and compare the potential of the CT-based radiomics in identifying ccRCC, chRCC and RO groups, receiver operating characteristic (ROC) curve analysis, and the area under the ROC curve (AUC) with 95% confidence interval (CI), sensitivity, and specificity values were calculated. These data were applied to evaluate the effectiveness of the models on the training and testing sets. In order to understand how a single radiomics feature contributes to the prediction of the model, the value of each feature was calculated.

### Nomogram construction and evaluation

A nomogram was constructed based on the clinical factors and the representative Rad-score in the training set. The calibration curves were plotted to evaluate the calibration of the nomogram. The ROC and AUC were calculated to quantify the performance of the nomogram on the training and testing sets. Decision curve analysis (DCA) based on the clinical factors and radiomics features in the testing set was used to calculate the net benefits for a series of threshold probabilities and assess the clinical value of the nomogram.

## Statistical analysis

The Kolmogorov–Smirnov test (K-S test) was conducted to test the normality of data distribution. The continuous parameters were computed using the Analysis of variance (ANOVA) and post hoc testing was applied for the analysis of pairwise differences, while the categorical variables were assessed using the χ^2^ test. All statistical analyses were performed using SPSS (version 25, Chicago, IL, USA). A two-tailed p-value < 0.001 was considered statistically significant. The representative radiomics features were correlated with the pathological index CK7 using Pearson’s correlation coefficients. The statistical significance of the balanced accuracy was computed by the permutation test (iteration 1000 times). Feature selection and model construction were carried out on the Anaconda3 platform (http://www.anaconda.com) with “scikit-learn” package (scikit-learn.org) using Python version 3.7.4. The nomogram was constructed and evaluated using the R statistical software (version 4.1.2).

## Results

### Patient characteristics

The training cohort consisted of 123 patients (chRCC: 25 males and 27 females, mean age: 53.0 ± 11.1 years; RO: 11 males and 17 females, mean age: 58.0 ± 13.7 years; ccRCC: 23 males and 20 females, mean age: 55 ± 10.5 years). The testing cohort consisted of 57 patients (chRCC: 13 males and 10 females, mean age: 57.3 ± 9.7 years; RO: 4 males and 9 females, mean age: 59.4 ± 7.8 years; ccRCC: 10 males and 11 females, mean age: 54 ± 10.9 years) collected based on the stratified sampling method. No significant differences were detected in the age and gender in the two groups in both the training and testing cohorts.

### Performance of radiomics feature screening and models

A total of 396 radiomics features were extracted from each phase. After performing ICC, mRMR, and LASSO regression, the remaining features were as follows: CMP: 6 features; NP: 5 features; combination: 11 features. The best-tuned regularization parameter of LASSO regression by tenfold cross-validation and the representative radiomic features of the combination are shown in Additional file 1: S1 and Table 1.

**Table 1:** Statistical analysis of the representative radiomic features derived from the combination

Feature names	chRCC	RO	ccRCC	F-value/*p *value
HaralickCorrelation_angle135_offset7_NP	2.90E+08 ± 2.26E+08	1.24E+09 ± 1.88E+09	4.68E+07 ± 2.48E+07	11.49/***
Inertia_angle135_offset4_CMP	513.09 ± 398.40	1140 ± 636.53	340.58 ± 299.05	12.06/***
uniformity_CMP	0.43 ± 0.19	0.61 ± 0.09	0.36 ± 0.23	14.97/***
ClusterProminence_angle0_offset7_CMP	3.51E+07 ± 4.55E+07	9.12E+07 ± 5.63E+07	2.43E ± 0.7 ± 5E+07	8.97/***
sumVariance_CMP	0.04 ± 0.02	0.06 ± 0.02	0.02 ± 0.02	21.15/***

^***^Denotes statistical significance, *p* < 0.001

**Fig. 3:**
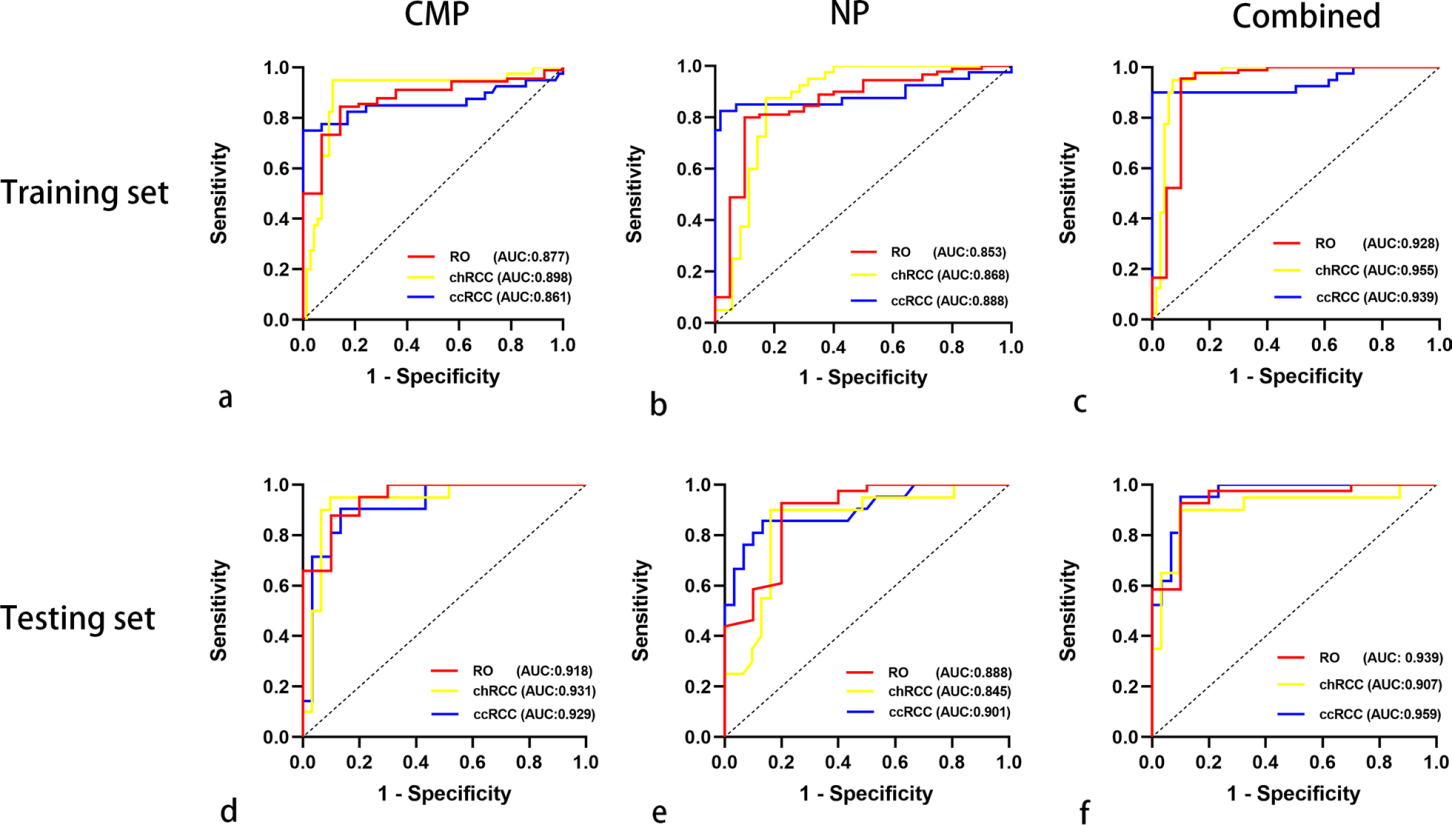
Comparison of ROC curves among CMP, NP, and combined models in the training (**a**–**c**) and testing sets (**d**–**f**)

**Fig. 4:**
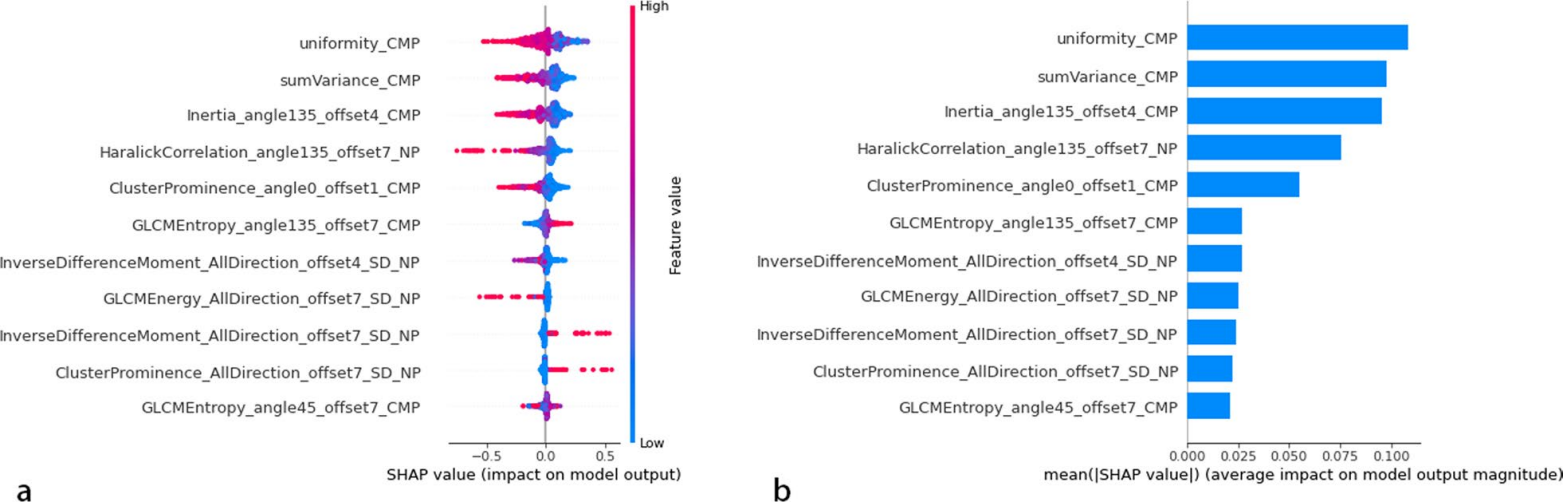
Summary plot of the impact features on the prediction of the SVM model. SHAP values of features in every sample. Each line represents a feature, and each dot represents a sample (**a**). The mean absolute value of the feature weight (**b**)

Figure 3 shows the AUCs of triple-class SVM models in the CMP and NP combination for RO, chRCC and ccRCC yielded values of 0.928 (95% CI 0.838–0.997), 0.955 (95% CI 0.913–0.996), and 0.939 (95% CI 0.880–0.997) in the training set and 0.939 (95% CI 0.855, 0.997), 0.906 (95% CI 0.810, 0.998), and 0.959 (95% CI 0.911, 0.996) in the testing set. Tables 2 and 3 listed the performance of the three classifiers. The SHAP values of the selected feature for each prediction were computed, and the SHAP of the combination is shown in Fig. 4.

**Table 2:** The diagnostic performance of the radiomic models in the training set (n = 123)

Classifier evaluation	CMP			NP			Combination		
	RO	chRCC	ccRCC	RO	chRCC	ccRCC	RO	chRCC	ccRCC
Average AUC	0.864	0.898	0.871	0.853	0.865	0.884	0.928	0.955	0.939
95% CI	0.792, 0.937	0.828, 0.968	0.780, 0.962	0.724, 0.933	0.797, 0.933	0.800, 0.969	0.838, 0.997	0.913, 0.996	0.880, 0.997
Balanced Accuracy	0.822	0.918	0.863	0.845	0.832	0.900	0.928	0.940	0.943
Average Sensitivity	0.844	0.950	0.775	0.789	0.850	0.825	0.956	0.950	0.900
Average Specificity	0.800	0.886	0.971	0.900	0.814	0.957	0.900	0.929	0.986

AUC, the area under the curve. CI, confidence interval. CMP, corticomedullary phase. NP, nephrographic phase

**Table 3:** The diagnostic performance of the radiomic models in the testing set (n = 57)

Classifier evaluation	CMP			NP			Combination		
	RO	chRCC	ccRCC	RO	chRCC	ccRCC	RO	chRCC	ccRCC
Average AUC	0.949	0.953	0.929	0.888	0.845	0.901	0.939	0.906	0.959
95% CI	0.878, 0.996	0.891, 0.998	0.859, 0.998	0.764, 0.997	0.730, 0.960	0.811, 0.991	0.855, 0.997	0.810, 0.998	0.911, 0.996
Balanced Accuracy	0.877	0.943	0.886	0.864	0.870	0.862	0.914	0.902	0.926
Sensitivity	0.854	0.950	0.905	0.927	0.900	0.857	0.927	0.900	0.952
Specificity	0.900	0.935	0.867	0.800	0.839	0.867	0.900	0.903	0.900

AUC, the area under the curve. CI, confidence interval. CMP, corticomedullary phase. NP, nephrographic phase

### Development and validation of the nomogram

**Fig. 5:**
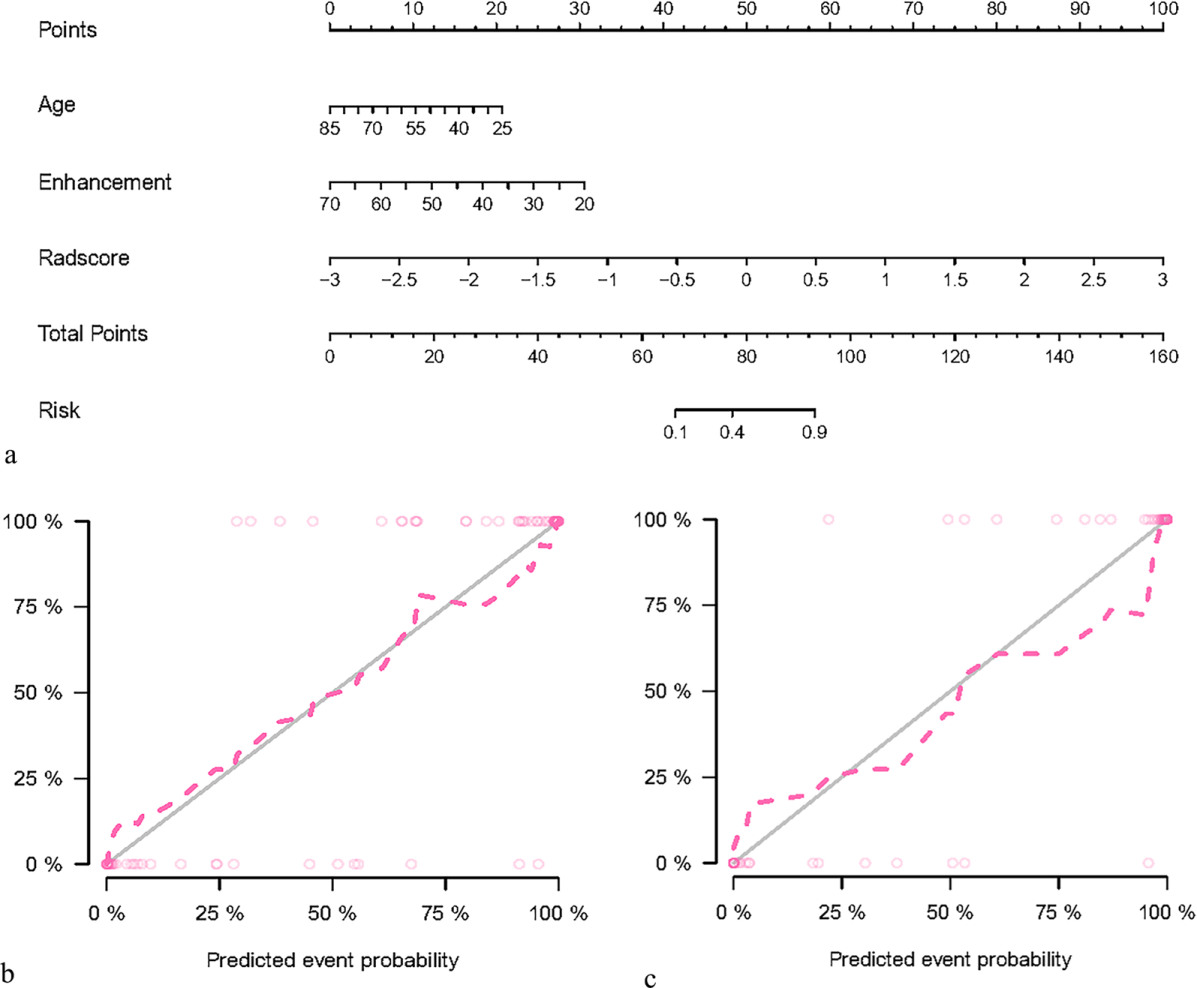
A radiomics nomogram incorporating the clinical feature, and a radiomics signature was developed in the training set (**a**). Calibration curves of the radiomics nomogram were used in the training set (**b**) and testing set (**c**). The y-axis represents the actual renal cell carcinoma rate, and the x-axis represents the predicted renal cell carcinoma possibility

**Fig. 6:**
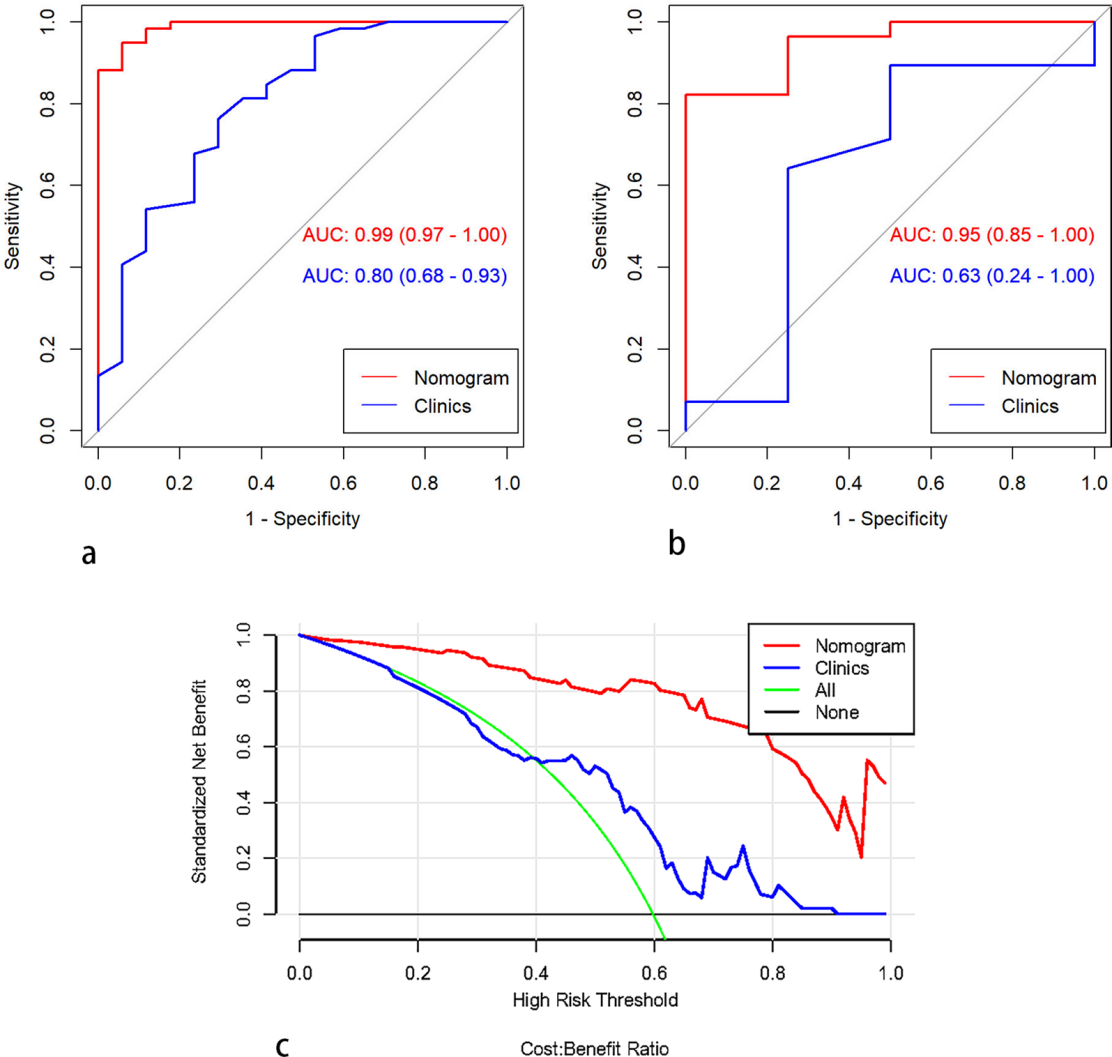
ROC curves of clinical and radiomics nomogram models in the training (**a**) and testing dataset (**b**). Decision curve analysis of the prediction models in the testing set (**c**). The y-axis measures the net benefit. The red line represents the radiomics nomogram. The green dotted line represents the assumption that all patients were renal cell carcinoma. The blue line represents the clinical prediction model. The red dotted line represents the radiomics model

The age, enhancement, and the radiomics features were included as independent predictors in the clinical radiomics nomogram, presented in Fig. 5a. The calibration curves showed good calibration in both the training and testing cohorts (Fig. 5b, c). The diagnostic performances of the clinical factor model and radiomics nomogram are presented in Table 4. The ROC curves for the models in the training and testing sets are shown in Fig. 6a, b. The DCA for the radiomics nomogram and clinical prediction model is presented in Fig. 6c. The radiomics nomogram showed a greater net benefit over the clinical model in differentiating ROs from chRCC and ccRCC in the testing set.

**Table 4:** The diagnostic performance of the nomogram in both the training and testing sets

	Training set		Testing set	
	Nomogram	Clinics	Nomogram	Clinics
AUC (95%CI)	0.990(0.970, 1.000)	0.800(0.680, 0.930)	0.950 (0.850, 1.000)	0.630 (0.240, 1)
ACC (95%CI)	0.947 (0.870, 0.985)	0.750(0.637, 0.842)	0.906 (0.750, 0.980)	0.781 (0.600, 0.907)
Sensitivity	0.949	0.763	0.931	0.821
Specificity	0.941	0.706	0.910	0.510

### Representative radiomics feature analysis in the combination phases

After assembling the LASSO regression and SVM, representative radiomics features were identified in the combination phases, including one histogram, two textural parameters, and one GLCM parameter. The radiomics signature and score were established by the following formula: Radscore = − 0.792*histogramEnergy_CMP + 1.013*HaralickCorrelation_angle135_offset7_NP-0.797*HaralickCorrelation_angle135_offset7_CMP-1.362*HighIntensityLargeAreaEmphasis_NP-1.132*Inertia_angle0_offset7_CMP-1.901*ClusterShade_AllDirection_offset1_SD_NP-0.89*Compactness2_CMP + 0.14*LargeAreaEmphasis_NP + 3.23.

**Fig. 7:**
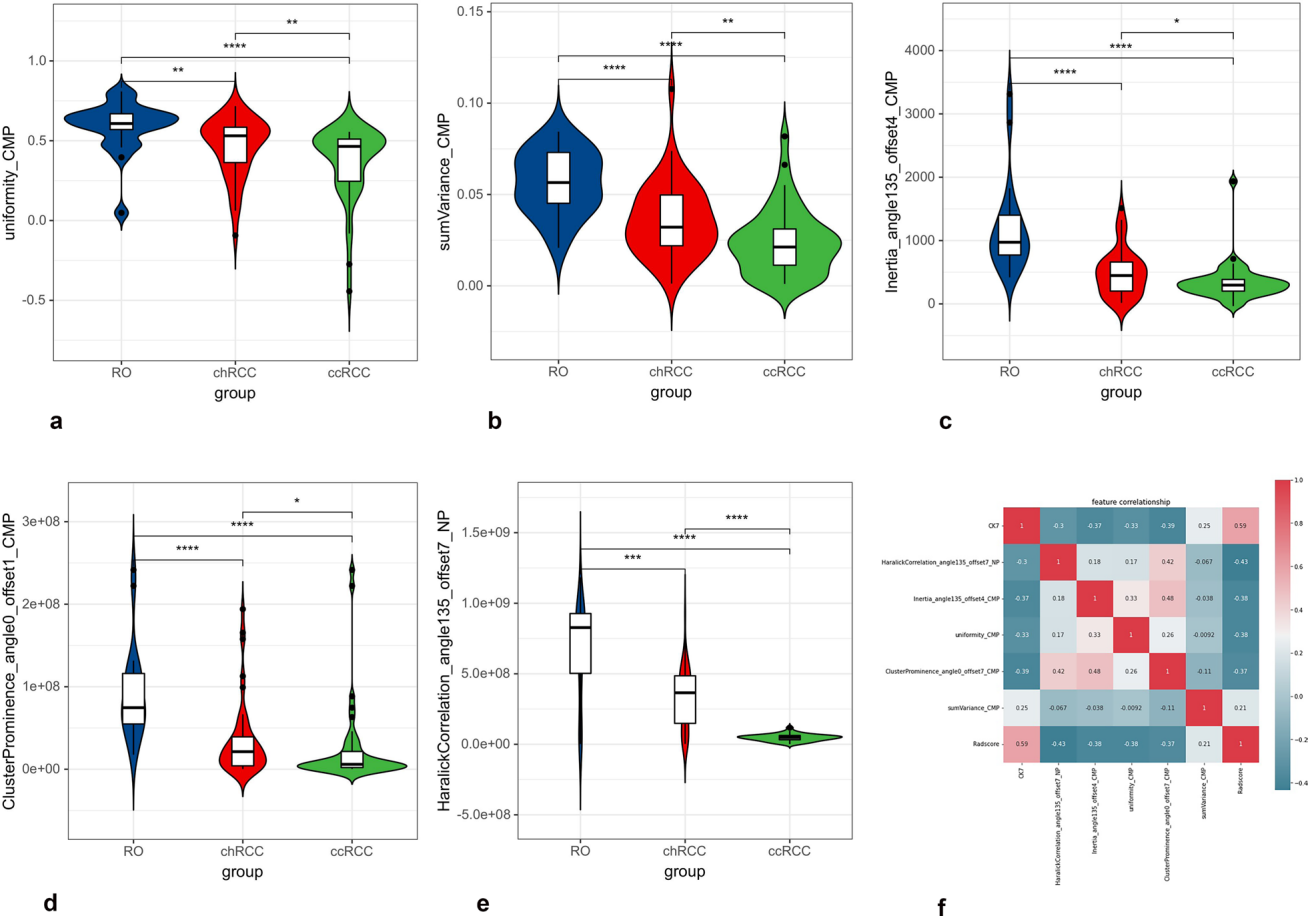
Distribution of representative radiomics features and the post-hoc statistical results in the three groups (**a**–**e**). Pearson’s correlation coefficient heatmap of mutual analysis between the representative radiomics features and clinicopathologic protein (**f**). The values in the square lattices represent the magnitude of the r values of the correlation analysis displayed by color differences

Figure 7 shows the results of the representative radiomics features. The histogram of the uniformity (0.61 ± 0.09 in RO; 0.43 ± 0.19 in chRCC, 0.36 ± 0.23 in ccRCC, *p* < 0.001) in RO patients was highest and lowest in ccRCC (Fig. 7a). The feature- sumVariance (0.06 ± 0.02 in RO; 0.04 ± 0.02 in chRCC, 0.02 ± 0.02 in ccRCC, *p* < 0.001) was highest in RO and lowest in ccRCC patients (Fig. 7b). The texture features Inertia_angle135_offset4 (1140 ± 636.53 in RO; 513.09 ± 398.40 in chRCC, 340.58 ± 299.05 in ccRCC, *p* < 0.001) and ClusterProminence_angle0_offset7 (9.12E+07 ± 5.63E+07 in RO; 3.51E+07 ± 4.55E+07 in chRCC, 2.43E ± 0.7 ± 5E+07 in ccRCC, *p* < 0.001) were highest in RO patients compared to the chRCC and ccRCC patients (Fig. 7c, d). The GLCM feature- HaralickCorrelation_angle135_offset7 (1.24E+09 ± 1.88E+09 in RO; 2.90E+08 ± 2.26E+08 in chRCC, 4.68E+07 ± 2.48E+07 in ccRCC, *p* < 0.001) was higher in RO than in chRCC and ccRCC patients (Fig. 7e).

Furthermore, Pearson’s correlation coefficient of CK7 and radiomics features are shown in Fig. 7f. CK7 was significantly correlated with uniformity, Inertia_angle135_offset4, ClusterProminence_angle0_offset7, HaralickCorrelation_angle135_offset7 and sumVariance (*p* = 0.007, r = -0.331; *p* = 0.002, r =  − 0.371; *p* = 0.002, r =  − 0.386; *p* = 0.016, r =  − 0.298, *p* = 0.02, r = -0.33 respectively), and especially with the Rad-score (*p* < 0.001, r = 0.594).

## Discussion

In the current study, we developed and validated a radiomics model based on the CT images from CMP and NP for a non-invasive distinction between RO and Renal Cell Carcinoma subtypes, which exhibited good performance. With the representative radiomics and clinical factors, a visual nomogram demonstrated an impressive efficiency with AUC of 0.91 in the testing set. What’s more, we found that the non-invasive radiomics factors has the ability of predicting the molecular protein CK7, which is important for accurate diagnosis and provide a promising molecular target for precise therapy.

In the present study, the value of histogram parameter-uniformity of RO was significantly higher than that of chRCC, which could be attributed to dispersed grayscale on CT images in malignant behaviour. The textural parameter-Cluster Prominence represented the pixel spatial distribution heterogeneity within an ROI. A higher cluster prominence value indicated an uneven distribution of the gray value in the ccRCC patients. This finding indicated that ccRCC is the most malignant renal tumor compared to chRCC and RO [25]. The textural parameter- Inertia reflected the texture groove depth of the image. The contrast is proportional to the texture groove. The value of Inertia was highest in RO and lowest in ccRCC patients, suggesting heterogeneous tumor tissues in ccRCC patients. We also found that the sumVariance is also related to the pathology grade. For pathological grade, RO are localized to inert lesions with noninvasive biological behaviour. The GLCM parameter-Haralick Correlation represents the correlation value of the local grayscale image and is used to measure the similarity of the grayscale image in the row or column [3]. We also found that the value of Haralick Correlation was highest in RO and lowest in ccRCC patients, suggesting a significant disorder of gray level in ccRCC patients. This result was in line with the physiological behavior of the tumors, as described previously [26]; the higher the degree of malignancy, the lower the value of Haralick-related parameters. Some studies have confirmed that the Haralick parameter is an index of reliability in texture analysis [27, 28]. Accordingly, the GLCM parameter-Haralick Correlation can avoid a large computational burden in the process of texture extraction. These results suggested that the physiological characteristics of the tumor tissue are complex in ccRCC patients. In this study, the radiomics features are utilized as an objective approach to assess the characteristics of carcinoma in clinical practice.

LASSO and SHAP algorithm describe the internal characteristics of the tumor. Herein, we applied the SVM classifier for an automated distinction among ccRCC, chRCC and RO. SVM has been applied to various body systems in medical images [29]. Several studies have focused on the application of machine learning-aided approaches for the diagnosis of renal tumors. In addition, applying a classifier further improves the performance of portal venous phase CT texture features for the differentiation of various RCC subtypes and oncocytoma [30]. However, the study did not eliminate redundancy. Conversely, the parameters of SVM in our study were selected by the “Grid Search CV” algorithm according to the best performance of the ten-cross validation, and a permutation test was used to confirm the learning efficiency. We found that the combination-phase model had the best performance with an average AUC of 0.941 and 0.935 in the training and testing sets, respectively, which was consistent with previous studies [31, 32]. This result may be due to the diversification of parameter characteristics, which improves the accuracy of the machine model for disease diagnosis.

Furthermore, the clinical and radiologic indicators associated with the malignant behavior of chRCC were also included in this study. Our radiomics nomogram may also increase the efficacy of distinguishing chRCC, ccRCC and RO in the training and testing sets. The DCA revealed that the radiomics nomogram could be clinically applicable. In addition, our study is the first report on the correlation between the radiomics features and the renal molecular protein. Pearson’s correlation coefficient was significant (*p* < 0.05) between the radiomics features and CK7 expression since CK7 is involved in tumorigenesis and associated with progression of chRCC. The radiomics features, extracted from the whole tumor and representing the physiology, could be used to non-invasively predict CK7 expression. Interestingly, in routine clinical work, when clinicians faced the challenge for RO, chRCC and chRCC,, the non-invasive radiomics could help accurate diagnosis and provide a promising molecular target for chRCC precise therapy.

## Generalizability issues and limitations

This study has several limitations. First, the sample size was relatively small, which could be attributed to the low clinical incidence of chRCC and RO. Second, it was a single-center, retrospective analysis, and thus the generalizability is subject to certain considerations. Hence, this radiomics-based method needs to be further verified by multicenter studies.

## Conclusions

In conclusion, we proposed a non-invasive and individualized CT-based radiomics nomogram to differentiation among RO, chRCC and ccRCC preoperatively and predict the immunohistochemical protein expression for accurate clinical diagnosis and treatment decision.

## Supplementary Information

**Additional file 1: Table S1**. The optimization parameters of the Lasso and SVM.

## Data Availability

The data that support the findings of this study are available from the First Affiliated Hospital of China Medical University but restrictions apply to the availability of these data, which were used under license for the current study, and so are not publicly available. Data are however available from the corresponding author upon reasonable request and with permission of the First Affiliated Hospital of China Medical University.

## Abbreviations

chRCC: Chromophobe renal cell carcinoma
ccRCC: Clear cell carcinoma
RO: Renal oncocytoma
CMP: Corticomedullary phase
NP: Nephrographic phase
ROI: Region of interest
VOI: Volume of interest
LASSO: Least absolute shrinkage and selection operator
SVM: Support vector machine
SHAP: Shapley additive explanations
GLSZM: Gray level size zone matrix
GLCM: Gray level cooccurrence matrix
RLM: Run length matrix
