# Supplementary material for: A triple-classification for differentiating renal oncocytoma from renal cell carcinoma subtypes and CK7 expression evaluation: a radiomics analysis
Source: BMC Urol. 2022 Sep 12;22:147. doi: 10.1186/s12894-022-01099-0 (PMC9469588; doi:10.1186/s12894-022-01099-0)
Supplement: Supplementary file 1 — Additional file 1: Table S1. The optimization parameters of the Lasso and SVM. [file 12894_2022_1099_MOESM1_ESM.docx]

Table S1

The optimization parameters of the Lasso and SVM

| Optimization Parameter | CMP | NP | Combination |  |
| --- | --- | --- | --- | --- |
| Lasso-λ | 3.76E-02 | 6.58E-02 | 1.03 E-02 | |
| SVM-C | 2.02E-02 | 2.33 | 9.26E-02 | |
| SVM-gamma | 9.26E-01 | 3.39E-02 | 7.20E-03 | |

CMP, corticomedullary phase. NP, nephrographic phase.
